# IFN‐γ and IL‐10 Immunosensor with Vertically Aligned Carbon Nanotube Interdigitated Electrodes toward Pen‐Side Cattle Paratuberculosis Monitoring

**DOI:** 10.1002/gch2.202400021

**Published:** 2024-08-25

**Authors:** Shaowei Ding, Benjamin J. Brownlee, Kshama Parate, Cicero C. Pola, Bolin Chen, Jesse M. Hostetter, Douglas Jones, John Jackman, Brian D. Iverson, Jonathan C. Claussen

**Affiliations:** ^1^ Mechanical Engineering Department Iowa State University Ames IA 50011 USA; ^2^ Department of Mechanical Engineering Brigham Young University Provo UT 84602 USA; ^3^ Department of Pathology Veterinary Medicine School Iowa State University Ames IA 20011 USA; ^4^ Department of Industrial and Mechanical Engineering Iowa State University Ames IA 50011 USA

**Keywords:** bovine disease, carbon nanotubes, electrochemical biosensor, Johne's disease, pen‐side monitoring

## Abstract

Highly sensitive vertically aligned carbon nanotube arrays (VANTAs) interdigitated electrode (IDE) arrays are developed for electrochemical biosensing of two cytokines (i.e., interleukin‐10 (IL‐10) and interferon‐gamma (IFN‐γ)) that are useful for early detection Johne's disease (Bovine Paratuberculosis) in cattle. The high aspect ratio VANTA‐IDEs (50–60 µm in height) are grown through a chemical vapor deposition process from an iron (Fe) catalyst that is lithographically patterned on a silicon wafer with equal finger width and inter‐finger spacing of 25 µm. After functionalization with distinct antibodies the VANTA‐IDEs are capable of selective detection of both IL‐10 and IFN‐γ within an actual biological matrix (i.e., diluted bovine implant supernatant) over concentration ranges of 0.1 to 30 pg mL^−1^ (limit of detection – LOD: 0.0911 pg mL^−1^) and 50–500 pg mL^−1^ (LOD: 24.17 pg mL^−1^), respectively with a response time of <35 min. Results demonstrate important initial steps for rapid, pen‐side identification of cattle with stage‐I *Mycobacterium avium* subspecies *paratuberculosis* infection before physical symptoms of Johne's disease are present. Such a rapid pen‐side diagnostic test can be used on cattle at an auction or before they are introduced to a herd to ensure the larger population does not become infected with Johne's disease.

## Graphical abstract

Vertically aligned carbon nanotube arrays (VANTAs) interdigitated electrode (IDE) arrays are capable of electrochemically monitoring both interleukin‐10 (IL‐10) and interferon‐gamma (IFN‐γ) concentrations within the bovine implant serum over a range of 0.1 to 30 pg/mL and 50‐1000 pg/mL, respectively, with a response time of <35 min.

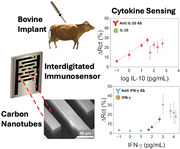

## 1 Introduction

There is a critical need for the development of an accurate diagnostic test for the identification of animals in the early infection stages (stage‐I) of *Mycobacterium avium* subspecies *paratuberculosis* (MAP).^[^ ^1^ ^]^ This pathogen is the causative agent of Johne's disease in cattle. Early (Stage‐I) detection of MAP is challenging as cattle do not yet exhibit serological markers, are not shedding the pathogen in feces, or exhibit detectable clinical signs such as diarrhea, weight loss, and debilitation.^[^ ^2^ ^]^ Detection of stage‐I‐infected cattle is advantageous because this is typically before they are shedding large numbers of bacteria into the environment. However, in later stages, infected cattle are highly contagious as they serve as the source of bacteria that can be spread to non‐infected calves through ingestion of infected manure or colostrum/milk. Current diagnostic assays for MAP detection are generally based on three distinct strategies: 1) identification of the bacterium directly, 2) identification of circulating immune‐response antibodies, or 3) monitoring cell‐mediated immunity. Monitoring bacteria in fecal matter or the monitoring of circulating antibodies in blood/serum samples are associated with late‐stage MAP infection where the animals that have contracted the disease are already a source of infection to non‐infected calves.^[^ ^2^ ^]^ As cell‐mediated immune responses are the first to develop during early MAP infection, MAP detection by monitoring cell‐mediated immunity is suited for the identification of stage‐I‐infected cattle.^[^ ^2^ ^]^ Current detection strategies for stage‐I MAP include measuring skin swelling in response to intradermally injected mycobacterial antigens (purified protein derivatives or PPD) or by detecting cytokines from ex vivo stimulated peripheral blood cells.^[^ ^3^, ^4^ ^]^ However, these detection strategies lack specificity to MAP and are prone to false positives from closely related environmental species of mycobacteria and vaccinated animals.^[^ ^5^ ^]^ Moreover, ex vivo assays require the use of blood samples that must be drawn from the cattle and then shipped to a lab where leukocytes are stimulated ex vivo and cytokines are measured. The lag time associated with shipping samples and the effect of artificial culture conditions on isolated bovine cells also increases the error and variability of the assay. Hence the use of a cell‐mediated immune response for MAP diagnostics in cattle has not been widely implemented. As a result, an in‐field, rapid, and cost‐effective assay that can detect MAP at stage I is greatly needed to reduce the spread of MAP throughout cattle herds. For example, such a pen‐side diagnostic test could be implemented at cattle auctions to ensure that animals are MAP‐free prior to purchase.

Currently, a stage‐I MAP biosensor does not exist. However, according to our previous work, minimally invasive monitoring of cytokine (interferon‐gamma (IFN‐γ)) using a collagen‐based implant would be a feasible approach to pen‐side detection of stage‐I MAP detection.^[^ ^6^ ^]^The detection of IFN‐γ from ex vivo stimulated blood leukocytes is a validated diagnostic assay for the detection of tuberculosis in humans and cattle.^[^ ^7^, ^8^ ^]^ Moreover, our group has identified both IFN‐γ and IL‐10 as key cytokines that are expressed in cattle tissues and blood during the early stages of MAP infection.^[^ ^9^ ^]^ Based on this work, we propose that measuring multiple cytokines in a MAP diagnostic platform will increase the accuracy of the test. Interest in using multiplexed cytokine detection approaches for the diagnosis of mycobacterial diseases has increased.^[^ ^7^, ^10^ ^]^ Recent research has shown that the evaluation of multiple cytokines rather than a single cytokine enhances the accuracy of these assays and provides important biomarkers of disease progression.^[^ ^11^ ^]^ Specifically, the ratio of IFN‐γ and IL‐10 has been successfully used for the diagnosis and prediction of disease outcomes in humans with tuberculosis^[^ ^12^ ^]^ and is indicative of cytokine components during the early immune response to MAP in cattle.^[^ ^13^ ^]^ Using a novel testing platform, which employs a removable subcutaneous collagen implant, we have detected high levels of both IFN‐γ and IL‐10 levels within tissues of MAP‐infected and vaccinated cattle.^[^ ^14^ ^]^

A variety of biosensing techniques exist for IL‐10 and IFN‐γ monitoring in biological fluids. Conventional laboratory techniques include sandwich enzyme‐linked immunosorbent assay (ELISA), enzyme‐linked immunospot assay (ELISPOT), reverse transcriptase polymerase chain reaction (RT‐PCR), flow cytometry, and surface plasmon resonance.^[^ ^13^, ^15^, ^16^, ^17^, ^18^, ^19^, ^20^ ^]^ Although these techniques can detect IFN‐γ and IL‐10 in low, biologically relevant concentrations (e.g., 1–25 pg mL^−1^), a number of common drawbacks (e.g., long detection times, extensive sample handling, manipulation of the cytokine, and requirement of expensive and/or bulky equipment) have restricted their use to the laboratory/clinical setting.^[^ ^21^ ^]^ Hence, several researchers have focused on the development of potential alternatives for IFN‐γ and IL‐10 detection, including field effect transistor biosensors (Bio‐FETs),^[^ ^22^, ^23^ ^]^ Förster resonance energy transfer (FRET) aptamer beacons,^[^ ^24^, ^25^, ^26^, ^27^, ^28^ ^]^ and localized surface plasmon resonance (SPR)‐based microfluidic optical devices^[^ ^27^ ^]^ and electrochemical biosensors. These biosensing techniques exhibit promise toward sensitive and rapid IFN‐γ and IL‐10 sensing, with detection limits extending down to the nano‐ and pico‐molar regimes.

Among the electrochemical strategies for cytokine detection, impedance‐based biosensors offer simple functionalization procedures, label‐free operation, and rapid response (<35 min), effective cost, and potential for in‐field applications.^[^ ^26^, ^29^, ^30^ ^]^ Moreover, the use of interdigitated electrodes (IDEs) as a platform for this type of biosensors offers advantageous mass transport and reaction kinetics (nonplanar or radial diffusion to each individual microband or IDE finger), higher current densities (electrical field lines concentrated around tightly packed microbands), and favorable Faradaic‐to‐capacitive current ratios. These benefits, associated with effective antibody‐antigen binding events, lead to improved signal‐to‐noise, higher sensitivity, and lower LOD.^[^ ^16^, ^31^, ^32^, ^33^ ^]^ The application of electrochemical biosensors for cytokines detection has been demonstrated in the literature, with sensing ranges of 1–15^[^ ^31^, ^32^ ^]^ and 0.1‐20 pg mL^−1[^ ^20^ ^]^ for IL‐10 and 1–5 ng mL^−1[^ ^16^ ^]^ and 0.0001–0.1 ng mL^−1[^ ^34^ ^]^ for IFN‐γ. However, these reports primarily focus on monitoring one cytokine type in humans and often require labeling steps for signal amplification, a challenging strategy for a pen‐side sensing paradigm.

Herein, we report the first 3D vertically aligned nanotube array (VANTA)^41[^ ^35^ ^]^ impedimetric immunosensor for label‐free detection of bovine IL‐10 and IFN‐γ cytokines. The VANTA IDEs, comprised of 20 interdigitated finger pairs of 25 µm width, were functionalized with Abs specific to bovine IL‐10 and IFN‐γ, and tested in PBS buffer as well as diluted bovine implant serum specifically acquired from cattle. The VANTA IDEs were capable of monitoring both IL‐10 and IFN‐γ concentrations within the bovine implant serum over a range of 0.1 to 30 pg mL^−1^ (LOD: 0.077 pg mL^−1^) and 50–1000 pg mL^−1^ (LOD: 95.87 pg mL^−1^), respectively with a response time of <35 min. Results demonstrate a significant first step in creating a biosensor capable of in‐field MAP detection for potential pen‐side bovine paratuberculosis diagnostics.

## 2 Experimental Section

### 2.1 Chemicals and Reagents

Potassium ferrocyanide, potassium ferricyanide, N‐(3‐Dimethylaminopropyl)‐N′‐ethylcarbodiimide (EDC), N‐hydroxy succinimide (NHS), ethanolamine, and FITC‐tagged anti‐mouse secondary Antibodies (Abs) were obtained from Millipore Sigma (Saint Louis, MO, USA). Mouse anti‐Bovine IL‐10 and IFN‐γ Abs and Bovine Antigens (Ags) were obtained from Bio‐rad (Bio‐Rad Laboratories, Inc., Hercules, CA, USA). Phosphate buffered saline (PBS), potassium chloride (KCl), and 2‐(N‐morpholino) ethanesulfonic acid (MES buffer, 0.5 m, pH 6.0) were purchased from Fisher Scientific (Hampton, NH, USA).

### 2.2 VANTA IDE Fabrication

The fabrication of high‐aspect‐ratio VANTA IDEs was performed by a chemical vapor deposition (CVD) process similar to previous reports.^[^ ^35^, ^36^ ^]^ Briefly, a 50 nm Al_2_O_3_ film was deposited onto an oxidized silicon wafer (1 µm oxide) using a Denton e‐beam evaporator. Photoresist (AZ 3330) was spun onto the wafer and developed to form the pattern of 40 IDE electrode fingers (20 pairs) of 25 µm spacing and width, each with a length of 1.95 mm. A 7‐nm Fe layer was thermally evaporated onto the wafer as a catalyst for carbon nanotube growth within a CVD furnace. The Fe was patterned for subsequent VANTA growth using a photoresist lift‐off procedure by stripping the photoresist with N‐Methyl‐2‐pyrrolidone for 10 min. VANTAs were CVD‐grown from the Fe catalyst by placing the metalized wafer (diced) in a Lindberg/Blue M tube furnace at 750 °C for 30 s while hydrogen and ethylene gas were pumped into the furnace at flow rates of 311 and 338 sccm, respectively. The resulting VANTA‐IDEs were approximately 50–60 µm in height. The VANTA structure was strengthened, to prevent collapse during electrochemical sensing, by coating the CNTs with amorphous carbon during infiltration by flowing hydrogen and ethylene (311 and 193 sccm, respectively) into the tube furnace at 900 °C for 3 min.^[^ ^37^ ^]^ This carbon infiltration conformally covers the VANTAs and Al_2_O_3_ coated wafer between the IDE fingers. This electrically conductive carbon floor is removed to prevent shorting across the IDE contacts using an oxygen plasma etch (Technics Planar Etch II) for 45 s at 250 W and 300 mTorr.

### 2.3 Bovine Implant Serum

**Figure 1:**
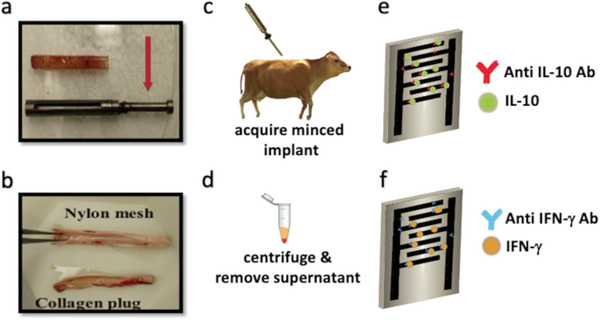
a) Stainless steel bovine implant with 4 cm × 0.5 cm sample body, 3 cm × 0.3 mm window, and 2 cm long pin (red arrow) for removal. b) Implant nylon mesh with a pore size of 30 µm surrounding a core of bovine purified collagen (PureCol, Advanced Biomatrix, Iowa City IA) placed in the stainless‐steel implant and exposed to cattle tissue through the window. c,d) The bovine implant is placed in the subcutis and removed after incubation. Implant material is removed from the housing, minced, and centrifuged to acquire supernatant. Schematic of functionalized e) IL‐10 and f) IFN‐𝛾 VANTA IDE sensors tested in diluted supernatant.

The bovine supernatant was obtained by an implant that was placed in the subcuits of the cattle (**Figure** **1**). The implant consists of a 4 cm × 0.5 cm stainless steel housing that has a single 3 cm × 0.3 mm window (Figure 1a). A 2‐cm solid pin was attached to one end of the implant to assist in removing the implant from the subcutis (Figure 1a, red arrow) The housing immediately surrounds a nylon mesh which was exposed to the exterior along the window in the housing (Figure 1b). The nylon mesh, with a pore size of 30 µm, surrounded a core of bovine‐purified collagen (PureCol, Advanced Biomatrix, Iowa City, IA). The implant with the nylon mesh and collagen was loaded into the neck of cattle by puncturing the skin (over the lidocaine bleb) such that it extended into the underlying subcutis. The trigger of the implant injection device was depressed, and the implant was delivered from the injection device into the subcutis. In this state, the nylon mesh allowed infiltration of immune cells and protein from the animal into the collagen but retained the collagen within the implant housing. After approximately 48 h the implant was removed and the bovine collagen within the nylon mesh was extracted for testing. This bovine collagen was processed by mincing and centrifuging within a polymer tube at a speed of 1000 RPM for 10 min. The supernatant from the tube was then removed and used for biosensing experiments conducted herein. It should also be noted that in this study the bovine collagen was acquired from a commercial source (Advanced Biomatrix, Iowa City, IA.) that uses pathogen‐free healthy cattle, and no measurable levels of the cytokines IL‐10 and IFN‐γ were detected in this collagen alone before implantation in the cattle. Figure 1e,f present a schematic view of the biofunctionalization and binding activity of IL‐10 and IFN‐γ Ab and Ag pairs.

### 2.4 Scanning Electron Microscopy (SEM)

Scanning electron microscopy (SEM) images of the VANTA IDEs were obtained using a FEI Quanta 250 field emission microscope (FEI Technologies, Hillsboro, OR, USA) at an accelerating potential of 5 kV.

### 2.5 Electrochemical Characterization

Electrochemical characterization was first performed in a 3‐electrode set‐up, including Ag/AgCl as the reference electrode, Pt wire as the counter electrode, and one of the two IDE finger arrays as the working electrode using a CHI6116E potentiostat workstation (CH Instruments, Austin, TX). A ferri‐/ferrocyanide (5 mM Fe(CN)_6_^3‐/4−^) redox probe with 0.1 m KCl was used for cyclic voltammetry (CV) measurements. Next, the impedance response was measured via non‐faradaic Electrochemical Impedance Spectroscopy (EIS) in the frequency range from 0.1 Hz to 1 MHz in a 2‐electrode set‐up. In this set‐up, increasing concentrations of KCl (10^−6^, 10^−5^, 10^−4^, 10^−3^, 10^−2^, 10^−1^, and 1 m) were added to 1X PBS while the impedance response of the VANTA IDEs was monitored.

### 2.6 Biofunctionalization Chemistry for Electrochemical Sensing

The activity of the Ag and Ab pair was tested prior to IDE functionalization using immunoblotting. VANTA IDEs were washed in 0.1 M MES buffer for 10 min, followed by an hour of incubation in a solution of 0.4 m EDC and 0.1 m NHS prepared in 0.1 m MES buffer. Devices were then washed in PBS, gently dried with nitrogen gas to remove excess liquid, and incubated overnight with 20 µg mL^−1^ of IL‐10 and IFN‐γ Abs. The reaction was terminated with 1.0 m ethanolamine for 20 min to quench unbound carboxyl groups. IDEs sensing surfaces were blocked in 2% BSA for 1 hour to reduce non‐specific binding of the Ags to bare areas of the electrode or substrate during electrochemical sensing. Finally, the functionalized electrodes were washed in PBS and dried with nitrogen gas before biosensing.

### 2.7 Biofunctionalization Chemistry for Fluorescence Microscopy

Two VANTA IDEs were prepared for fluorescence spectroscopy measurements as follows. A positive control electrode was biofunctionalized using the same Ab functionalization protocol as explained above. A second negative control electrode was prepared in a similar manner but without the use of primary Ab. Both positive and negative control IDEs were then incubated with 2% BSA for 1 h, to again help minimize non‐specific binding during experiments by blocking bare areas of the electrode or substrate during fluorescence imaging. Next, 10 µL of 10 µg mL^−1^ of anti‐mouse IgG‐FITC Ab was placed on the positive and negative control IDEs and allowed to incubate on the electrodes for approximately 1 h. Fluorescence is only visible when the FITC‐labeled secondary Ab binds to the primary Ab immobilized on the positive control electrode. The negative control, which does not contain primary Ab, was used to demonstrate that negligible FITC‐labeled secondary Ab was capable of non‐specifically binding to the VANTA IDEs.

### 2.8 IFN‐γ and IL‐10 Biosensing

After this initial electrochemical characterization, the VANTA IDEs were functionalized with anit‐IL‐10 or anti‐IFN‐γ Abs. These Ab functionalized sensors were then allowed to incubate with their target Ags in PBS or the actual complex biological matrix (i.e., bovine implant supernatant acquired as previously described), then rinsed with PBS, and then operated in a 2‐electrode set‐up for EIS measurements while being submerged in 5 mM Fe(CN)_6_^3‐/4‐^ PBS solution. This IDE sensing arrangement could be operated without a reference electrode and provided a simple means for obtaining a steady state current response and was more amenable to large‐scale fabrication, especially compared to configurations of three and four electrodes.^[^ ^38^ ^]^ Calibration curves were acquired by adding 10 µL of sequentially higher concentrations of Ags (0.1–10000 pg mL^−1^), incubating for 30 min, rinsing with PBS, and then performing EIS measurements over a frequency range of 0.1 Hz to 1 MHz with an AC amplitude of 5 mV. IL‐10 and IFN‐γ sensing tests were performed separately within 10 µL of PBS or within 10 µL of diluted cattle implant supernatant (1:1000). The stability and selectivity of the VANTA IDE biosensors were tested using different concentrations of similar cytokines (IL‐10, IFN‐γ, IL‐6) and BSA, as well as different incubation times.

### 2.9 Data Analysis

A completely randomized design was used in this study with at least three replicates, and the results were reported as mean ± standard deviation. Regression analysis was performed to determine the linear sensing range and the functional correspondence between the concentration of the target analyte and the change in charge transfer resistance with a confidence level of 95% (α = 0.05). JMP Pro v.17 statistical software (SAS Institute, Cary, NC, USA) was used for the statistical analysis. The limit of detection was calculated using the 3‐sigma method.^[^ ^39^ ^]^

## 3 Results and Discussion

**Figure 2:**
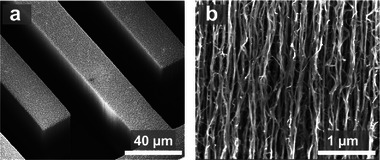
Scanning electron micrographs of the VANTA IDEs displaying a) the 3D structure of the IDE fingers (at 1,000x) and b) the vertically aligned carbon nanotube arrays (at 35,000x).

The VANTA IDEs were fabricated to a height of 50–60 µm using a chemical vapor deposition method similar to previous reports (see Experimental Section).^[^ ^36^, ^37^ ^]^ The IDE sensor was comprised of 2 finger combs containing 20 fingers each that measured 1.95 mm in length with 25 µm width and spacing, resulting in a geometric area of approximately 9.9 × 10^−3^ cm^2^. The VANTA IDE's highly porous and defect‐rich 3D structure is displayed in **Figure** **2**. The magnified image of the VANTA sidewall revealed the compacted matrix of vertically aligned carbon nanotubes (Figure 2b). The VANTAs were infiltrated with amorphous carbon for stability, resulting in a mechanically stable hydrophilic structure with a water contact angle of 69° and superficial carboxylic groups amenable to biofunctionalization.^[^ ^35^ ^]^ The carboxyl groups on the VANTA IDE were functionalized with a 20 µg mL^−1^ amine terminated Abs through EDC/NHS chemistry while the remaining unbound functional groups were blocked with a 2% bovine serum albumin (BSA) solution.

**Figure 3:**
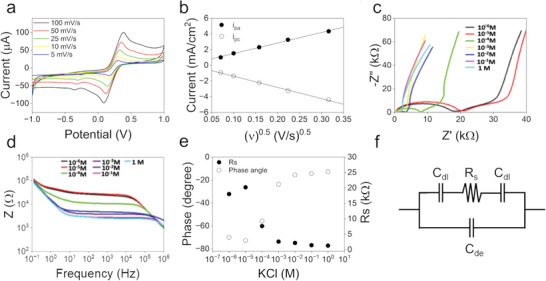
Electrochemical characterization of VANTA IDEs. a) CV measurements at different scan rates (5, 10, 25, 50, and 100 mV s^−1^). b) Current density versus the square root of the scan rate. (c‐d) EIS measurements acquired with various KCl concentrations (10^−6^, 10^−5^, 10^−4^, 10^−3^, 10^−2^, 10^−1^, and 1 m). e) Phase change plot at 100 kHz and *R_s_* plot at distinct concentrations of KCl. f) The equivalent circuit for the non‐faradaic EIS of the VANTA IDEs.

Functionalized VANTA IDEs were first electrochemically tested in 10 µL of PBS as a negative control and then 10 µL of diluted cattle implant supernatant (with a known concentration of cytokines) as described in the Experimental Section and Figure 1. CV and EIS measurements were acquired in KCl to measure the electrochemical reactivity of the VANTA‐IDEs. Well‐defined anodic and cathodic peak currents (*I_p_*) were obtained across a range of scan rates (5, 10, 25, 50, and 100 mV s^−1^) due to the ferricyanide, Fe^3+^/Fe^2+^ redox couple (**Figure** **3**a). The linear relationship between peak currents and the square root of the voltage scan rate demonstrated from the Randles‐Sevcik plot in Figure 3b is indicative of a reaction that was controlled by the diffusion of species in solution. Moreover, the peak‐to‐peak separation (ΔE_p_) values range from 0.12 V to 0.32 V, which is indicative of limitations in electron transfer, suggesting a quasi‐reversible system. Such ΔE_p_ values are significantly lower than those obtained during ferricyanide electrochemistry with electrodes comprised of basal planes of highly oriented pyrolytic graphite (ΔE_p_ 0.63 V).^[^ ^40^ ^]^ The ΔE_p_ values of the VANTA IDEs also compare favorably with similar CNT‐based electrodes where aligned MWCNTs electrodes achieved ΔE_p_ values of 0.23 V.^[^ ^40^ ^]^ The effective electroactive surface area (A = 8.9 × 10^−5^ cm^2^, geometric area ≈ 9.9 × 10^−3^ cm^2^) of the VANTAs was also calculated by incorporating the recorded I_p_ values into the Randles–Sevcik equation (Equation 1),

(1) $$I_{p}=\left(2.69\;\times \;10^{5}\right)\;n^{\frac{3}{2}}v^{\frac{1}{2}}D^{\frac{1}{2}}CA$$

where *n* is the number of electrons transferred by the Fe^3+^/Fe^2+^ redox couple (*n* = 1)*, v* is the scan rate*, D* is the diffusion coefficient (*D* = 7.20 × 10^−6^ cm^2^ s^−1^), and *C* is the analyte concentration (*C* = 5 mm). The surface area where heterogeneous charge transport occurs can be reported as a percent of active sites (the ratio between electroactive surface area and geometric area). The calculated electroactive surface area results in approximately 0.9% active sites for the VANTA IDEs in a Fe^3+^/Fe^2+^ redox couple. Such values are greater than previous reports of 0.4% active sites for single‐walled carbon nanotube networks immobilized in a planar fashion on electrodes.^[^ ^41^ ^]^ Thus, the porous and vertically‐aligned nature of the VANTA IDEs considerably raises the electroactive surface area above conventional solid or planar IDE sensors^[^ ^42^ ^]^ and provides more carbon‐carbon defects or active sites than conventional CNT electrodes.^[^ ^43^, ^44^ ^]^

To further electrochemically characterize the VANTA IDEs, non‐faradic EIS measurements were conducted with increasing concentrations of KCl (from 1.0 µm to 1.0 m KCl) (Figure 3c–e).

An equivalent circuit model, based on the work developed by Zou et al.,^[^ ^33^ ^]^ was used to help elucidate the non‐faradic EIS data acquired from the VANTA IDEs (Figure 3f). At lower frequencies and higher concentrations of KCl, the impedance response is predominantly dominated by the double‐layer capacitance (C_dl_), which is formed on the interface of the neighboring fingers and the electrolyte. Within the intermediate frequency range, the impedance is governed by *R*_s_, which arises from the changes in ionic concentration in the solution. Finally, at higher frequencies, the system impedance is influenced by the solution dielectric capacitance (C_de_), which has its effect reduced as the concentration of KCl increases, approaching a shortened stage at the higher levels of KCl.^[^ ^42^ ^]^ Similar impedance behavior at different concentrations of KCl was observed previously by Hardeman et al.^[^ ^45^ ^]^ and Zou et al.,^[^ ^33^ ^]^ validated that the conductivity of the solution is a determinant factor in constraining the signal of the sensor in the intermediate frequency range.^[^ ^45^ ^]^ As evidenced in Figure 3d, changes in the solution resistance (R_s_) are correlated with KCl concentrations between 1 and 100 µm, while the phase change was sensitive to KCl concentration changes varying between 100 µm and 1 m. Hence, the response to surface‐related processes can be maximized at specific concentrations of electrolyte and frequency ranges.^[^ ^46^ ^]^

**Figure 4:**
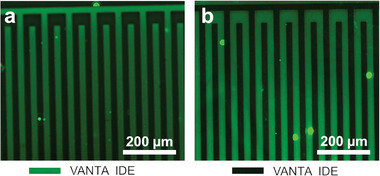
Fluorescent micrographs of the VANTA IDEs after anti‐mouse IgG‐FITC Ab was immobilized on VANTA IDEs and incubated for 1 hr. Resultant micrographs display binding of IgG‐FITC Ab onto VANTA IDEs that have been previously functionalized a) with anti‐ bovine IFN‐γ Ab (fluorescence observed on VANTA structures is shown in green) and b) without (no fluorescence observed on VANTA structures as they remain black).

Fluorescence microscopy was next performed to verify the effectiveness of the VANTA IDE antibody biofunctionalization protocol using FITC fluorescence tagged secondary Abs that bind to primary Ab‐Ag pairs covalently bound to the VANTA surface (see Experimental Section). **Figure** **4**a shows a positive fluorescence emission originating from the VANTA IDE fingers as the secondary FITC labeled Abs effectively bound the primary Abs on the VANTA surfaces. However, in the example of the negative control (Figure 4b), where no primary Ab has been conjugated onto the VANTA IDEs, the fluorescence emission areas were reversed and the VANTA IDEs did not fluoresce after incubation with the FITC labeled secondary Abs.

**Figure 5:**
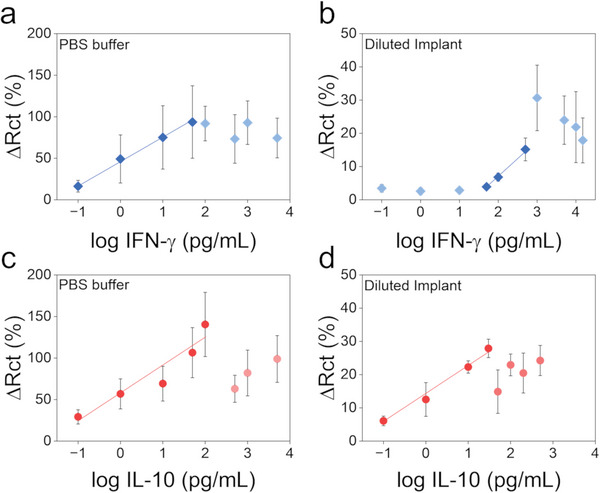
IFN‐γ sensing in a) PBS buffer with linear sensing range from 0.1 to 50 pg mL^−1^ (p_model_ = 0.0064; p_LOF_ = 0.984; R^2^ = 0.9965) and LOD of 0.0480 pg mL^−1^ and b) 1000x diluted cattle implant supernatant with linear sensing range from 50 to 500 pg mL^−1^ (p_model_ = 0.0002; p_LOF_ = 0.772; R^2^ = 0.998) and LOD of 24.17 pg mL^−1^). IL‐10 sensing in c) PBS buffer with linear sensing range from 0.1 to 100 pg mL^−1^ (p_model_ = 0.0001; p_LOF_ = 0.352; R^2^ = 0.8943) and LOD of 0.0793 pg mL^−1^ and d) 1000x diluted cattle implant supernatant with linear sensing range from 0.1 to 30 pg mL^−1^ (p_model_ = 0.0001; p_LOF_ = 0.597; R^2^ = 0.9571) and LOD of 0.0848). Data represents mean ± standard deviation Error bars represent one standard deviation from the mean forfrom three distinct experiments with biosensors (n = 3).

The VANTA IDE electrodes functionalized with either IL‐10 or IFN‐γ Abs were first calibrated through incubations in PBS buffer and next in diluted cattle implant (Figure 1 and Experimental Section). The Abs concentration used to functionalize the VANTA IDEs was diluted to 20 µg mL^−1^ as this concentration improved the sensing range of the VANTA IDEs over lower Abs concentrations, according to preliminary tests. This selected Ab concentration was covalently immobilized onto the VANTA IDEs using EDC/NHS chemistry with approximately a 35 min incubation time. Then, faradaic EIS measurements were performed in Fe^3+^/Fe^2+^ redox probe with 0.1 m KCl, and the Randles equivalent circuit was used to extract information about the changes in charge transfer resistance (Rct). As IFN‐γ and IL‐10 interact with the antibodies on the VANTA IDE, an insulating layer is formed on the electrode surface, resulting in Rct values.^[^ ^47^ ^]^ Then, a calibration plot correlating the measured Rct with the concentration of the cytokine tested is obtained. Subsequent IFN‐γ calibration tests in PBS displayed a linear sensing range from 0.1 to 50 pg mL^−1^ (p_model_ = 0.0064) with a LOD of 0.048 pg mL^−1^ (y = 28.401x + 46.507) while in diluted cattle serum yielded a linear sensing region of 50–500 pg mL^−1^ (p_model_ = 0.0002) with a detection limit of 24.17 pg mL^−1^ (y = 11.359x – 15.594) (**Figure** **5**a,b). Calibration plots of IL‐10 in PBS buffer (Figure 4c) yielded a linear sensing range from 0.1 −100 pg mL^−1^ (p_model_ = 0.0001) with a LOD of 0.0793 pg mL^−1^ (y = 33.133x + 56.285) while IFN‐γ sensing in diluted cattle implant (Figure 5d) resulted in a sensing range of 0.1 to 30 pg mL^−1^ (p_model_ = 0.0001) and a detection limit of 0.09112 (y = 8.9729x + 13.4853). Compared to the sensing result in PBS, sensing in cattle serum had a higher detection limit but a wider sensing range. This decrease in biosensor sensitivity within the cattle serum is most likely due to a decrease in Ab‐Ag binding which could have been hindered due to the complexity of the sample matrix with potential interferents found endogenously within this biological solution. All sensing curves presented a signal drop at higher concentrations of the target analyte. This behavior is an indication of biosensor saturation, which results from the imbalance between the recognition agent attached to the surface of the electrode and the high concentration of target analytes present in the sample. Then, the Ab‐Ag complex formed on the electrode surface disassociates and is released in the solution, resulting in a decrease in response signal.^[^ ^48^, ^49^ ^]^

The performance of the VANTA IDE immunosensors is relevant for the detection of IFN‐γ at the early stages of paratuberculosis, considering that levels between 1 and 10 ng mL^−1^ are reported in serum samples.^[^ ^50^ ^]^ Also, the sensor developed here could be successfully applied for the detection of average levels of IL‐10 (8 ng mL^−1^) in cattle with paratuberculosis.^[^ ^51^ ^]^ Furthermore, we observed an improvement in cytokine sensing performance for the VANTA IDEs when compared to a previously reported work on printed graphene IDEs. Parate et al.^[^ ^52^ ^]^ developed an aerosol jet printed (AJP) IDE from graphene‐nitrocellulose ink for the impedimetric detection of both IFN‐γ and IL‐10 in serum. Besides obtaining similar LOD results for IFN‐γ (AJP = 25 pg mL^−1^ and VANTA IDE = 24.17 pg mL^−1^), the VANTA IDEs (0.09112 pg mL^−1^) presented and LOD more the two orders of magnitude lower than the AJP IDE (46 pg mL^−1^). The VANTA IDE immunosensor results were also competitive with other considerably more complex sensing devices. For example, an interesting approach was presented by Januarie et al.^[^ ^53^ ^]^ while developing a sensor for IFN‐γ. These authors fabricated an aptasensor using metal dichalcogenide‐tin telluride selenide combined with quantum dots. This device presented an LOD of 0.151 pg mL^−1^ in buffered saline solution, which is considerably higher than the LOD obtained for the VANTA IDEs in the buffer. In another work, Saputra et al.^[^ ^54^ ^]^ applied conducting polymer composited with MWCNT and gold nanoparticles (AuNP) to develop a glassy carbon (GCE)‐based electrochemical aptasensor for IFN‐γ. Even though a commercial GCE was used together with MWCNT and AuNP, this device presented an LOD of 7.68 fg mL^−1^, a limited improvement compared to the VANTA IDE immunosensor. Similarly, Nanda et al. developed an immunosensor for IL‐10 using commercial glassy carbon electrodes decorated with AuNP. Frias et al.^[^ ^55^ ^]^ reported on a microfluidic device for impedimetric detection of IL‐10 using commercial screen‐printed electrodes modified with graphene foam. Besides the low LOD value observed (7.89 fg mL^−1^), this device presented limitations in the linear sensing range (10–100 fg mL^−1^). Notably, the VANTA IDEs immunosensors developed here presented competitive performance with similar electrochemical biosensors reported in the literature and could be easily tuned to target different cytokines by a simple change in the antibody used as a recognition agent.

**Figure 6:**
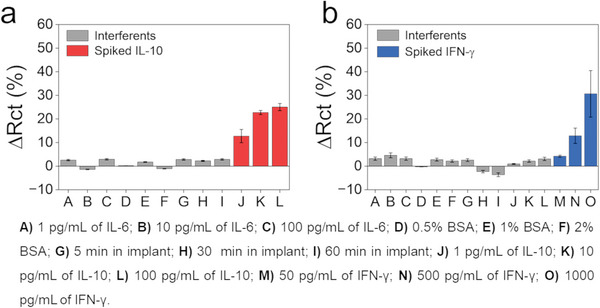
Interferent testing with IL‐6 (1, 10, and 100 pg mL^−1^), BSA (0.5%, 1%, and 2%), and increased incubation time (5, 30, and 60 min) when the VANTA IDE was functionalized with a) anti‐ IL‐10 Abs and b) anti‐ IFN‐γ Abs. Representative data detecting IL‐10 (at 1, 10, and 100 pg mL^−1^) and IFN‐γ (at 50, 500, and 1000 pg mL^−1^) are also displayed for comparison. Inset (b) also indicates the response to IL‐10 (1, 10, and 100 pg mL^−1^) for a sensor functionalized with IFN‐γ Abs.

Finally, to evaluate the selectivity and stability of the proposed sensing platform, the functionalized electrodes with anti‐ IL‐10 Abs and anti‐ IFN‐γ Abs were used to monitor the change in signal from sequentially monitoring similar cytokines (IL‐10 for the IFN‐γ biosensors and IL‐6 for the IL‐10 biosensor at concentrations of 1, 10, and 100 pg mL^−1^), distinct concentrations of BSA (0.5%, 1%, and 2%) and as well as increased incubation time of the 1000 times diluted cattle implant (5, 30, and 60 min). The results plotted in **Figure** **6** indicate that the percent change in charge transfer was minimal for all interferent species tested as compared to the signals obtained from the target analyte concentrations IL‐10 and IFN‐γ, respectively. Hence, these results indicate there was minimal non‐specific binding and the probability of false positive biosensor results, even within a complex biological solution, is expected to be low.

## 4 Conclusion

In conclusion, the presented sensing platform is the first use of VANTA IDEs for electrochemical biosensing of bovine IL‐10 and IFN‐γ. This work demonstrates the ability to detect cytokines, which can be used to indicate MAP disease status, and builds on previous work demonstrating a similar approach with oncoproteins.^[^ ^35^ ^]^ VANTA fabrication techniques were coupled with carbon infiltration to achieve highly ordered and vertically aligned carbon nanotube forests with significantly increased surface area in an IDE arrangement.^[^ ^37^ ^]^ Oxygen plasma etching was used to render the electrode surface hydrophilic and populated with carboxylic groups, making the VANTA IDE well‐suited for covalent Ab biofunctionalization via EDC/NHS chemistry.^[^ ^36^ ^]^ Functionalized IDEs were tested in PBS buffer and diluted cattle implant supernatant. IFN‐γ sensing with functionalized VANTA IDEs achieved a linear sensing range of 50–500 pg mL^−1^ with a detection limit of 24.17 pg mL^−1^, while for IL‐10, a linear sensing range of 0.1–30 pg mL^−1^ and a detection limit of 0.0848 pg mL^−1^ was achieved in diluted cattle implant supernatant. The detection of both cytokines was demonstrated in actual biological matrix, viz., diluted cattle implant supernatant, as opposed to previous works performed in PBS buffer and animal solutions not acquired from cattle. In future work, a full trail of diseased and healthy cattle will be examined with this bovine collagen implant – biosensor technology. During such an extensive study, it is expected that this sensor could help elucidate the immune response of cattle in different stages of MAP infection and fully characterize the cytokine profile of stage I animals. We envision an initial use of this novel diagnostic tool in screening cattle before they are introduced into a new herd. This would be a vital diagnostic tool for preventing the introduction of asymptomatic MAP‐infected cattle in herds that will inevitably be a source of MAP infection for the herd. The cost and time of such a full trial is out of the scope of this manuscript.

The developed VANTA IDE biosensor achieved label‐free detection of target analyte with minimal interference from similar cytokines (IL‐6, IL‐10 in the case of IFN‐γ sensing; IL‐6 in the case of IL‐10 sensing) and BSA. Additionally, a rapid detection time (<35 min) was achieved, promising for pen‐side or point‐of‐service sensing applications. The electrochemical sensing method utilized in this work, performed without sample enrichment or skilled personnel, is an appealing alternative to conventional lab techniques, which are time‐consuming, expensive, and labor‐intensive. Moreover, the resultant VANTA sensing platform can overcome the challenges related with fluorescence/optical based sensors that require complex equipment/processes (e.g., fluorescence microscopes, pre‐labeling steps), and does not suffer from problems related to stability and photobleaching of fluorophores. Consequently, it enables the possibility of functioning in turbid, optically dense, or autofluorescent biological samples—conditions that are typically found with field samples.^[^ ^56^ ^]^ Moreover, the fabrication protocol and material characterization results for 3D VANTA IDEs developed herein could be applied to emerging fields that have incorporated CNTs into numerous electrochemical applications including sensors, fuel cells, actuators, and energy harvesters.^[^ ^57^, ^58^, ^59^ ^]^

## Conflict of Interest

The authors declare no conflict of interest.

## Data Availability

The data that support the findings of this study are available from the corresponding author upon reasonable request.

## References

[1] National Research Council (US) Committee on Diagnosis and Control of Johne's Disease. Diagnosis and Control of Johne's Disease, National Academies Press, Washington, DC 2003. 25032299

[2] A. Tiwari , J. A. VanLeeuwen , S. L. McKenna , G. P. Keefe , H. W. Barkema , Can. Vet. J. 2006, 47, 874. 17017652 PMC1555680

[3] J. R. Stabel , K. Kimura , S. Robbe‐Austerman , J. Vet. Diagn. Invest. 2007, 19, 43. 17459831 10.1177/104063870701900107

[4] C. H. J. Kalis , M. T. Collins , J. W. Hesselink , H. W. Barkema , Vet. Microbiol. 2003, 97, 73. 14637040 10.1016/j.vetmic.2003.07.003

[5] E. Gormley , M. Doyle , A. Duignan , M. Good , S. J. More , T. A. Clegg , Vet. Res. 2013, 44, 117. 24308747 10.1186/1297-9716-44-117 PMC4028746

[6] B. L. Plattner , E. L. Huffman , J. M. Hostetter , Vet. Pathol. 2013, 50, 630. 23051915 10.1177/0300985812463404

[7] K. E. Bass , B. J. Nonnecke , M. V. Palmer , T. C. Thacker , R. Hardegger , B. Schroeder , A. J. Raeber , W. R. Waters , Clin. Vaccine Immunol. 2013, 20, 1827. 24132602 10.1128/CVI.00519-13 PMC3889511

[8] M. Denis , D. N. Wedlock , A. R. McCarthy , N. A. Parlane , P. J. Cockle , H. M. Vordermeier , R. G. Hewinson , B. M. Buddle , Clin. Vaccine Immunol. 2007, 14, 1483. 17881504 10.1128/CVI.00291-07 PMC2168177

[9] S. M. Albarrak , W. R. Waters , J. R. Stabel , J. M. Hostetter , Vet. Immunol. Immunopathol. 2018, 201, 26. 29914678 10.1016/j.vetimm.2018.05.003

[10] N. Nausch , C. Lundtoft , G. Schulz , H. Henckel , E. Mayatepek , B. Fleischer , F. M. Marx , M. Jacobsen , Int. J. Tuberc. Lung Dis. 2017, 21, 270. 28225337 10.5588/ijtld.16.0351

[11] E.‐J. Won , J.‐H. Choi , Y.‐N. Cho , H.‐M. Jin , H. J. Kee , Y.‐W. Park , Y.‐S. Kwon , S.‐J. Kee , J. Infect. 2017, 74, 281. 27871809 10.1016/j.jinf.2016.11.010

[12] K. H. Skolimowska , M. X. Rangaka , G. Meintjes , D. J. Pepper , R. Seldon , K. Matthews , R. J. Wilkinson , K. A. Wilkinson , PLoS One 2012, 7, e46481. 23071578 10.1371/journal.pone.0046481 PMC3468619

[13] T. Hussain , S. Z. A. Shah , D. Zhao , S. Sreevatsan , X. Zhou , Cell Commun. Signal. 2016, 14, 29. 27905994 10.1186/s12964-016-0152-z PMC5131435

[14] T. Lindquist , Evaluating the in Vivo Immune Response to Mycobacterium Avium Subspecies Paratuberculosis Infection in Naive and Vaccinated Calves, Iowa State University, Ames 2018.

[15] N. Favre , G. Bordmann , W. Rudin , J. Immunol. Methods 1997, 204, 57. 9202710 10.1016/s0022-1759(97)00033-1

[16] S. Ding , C. Mosher , X. Y. Lee , S. R. Das , A. A. Cargill , X. Tang , B. Chen , E. S. McLamore , C. Gomes , J. M. Hostetter , J. C. Claussen , ACS Sens. 2017, 2, 210. 28723140 10.1021/acssensors.6b00581

[17] E. Holler , M. G. Roncarolo , R. Hintermeier‐Knabe , G. Eissner , B. Ertl , U. Schulz , H. Knabe , H. J. Kolb , R. Andreesen , W. Wilmanns , Bone Marrow Transplant. 2000, 25, 237. 10673693 10.1038/sj.bmt.1702126

[18] J. D. Ohmen , J. M. Hanifin , B. J. Nickoloff , T. H. Rea , R. Wyzykowski , J. Kim , D. Jullien , T. McHugh , A. S. Nassif , S. C. Chan , R. L. Modlin , J. Immunol. 1995, 154, 1956. 7836775

[19] C. T. Campbell , G. Kim , Biomaterials 2007, 28, 2380. 17337300 10.1016/j.biomaterials.2007.01.047

[20] X. Jiang , M. G. Spencer , Biosens. Bioelectron. 2010, 25, 1622. 20047827 10.1016/j.bios.2009.11.024

[21] J. A. Stenken , A. J. Poschenrieder , Anal. Chim. Acta 2015, 853, 95. 25467452 10.1016/j.aca.2014.10.009 PMC4717841

[22] K. Min , M. Cho , S.‐Y. Han , Y.‐B. Shim , J. Ku , C. Ban , Biosens. Bioelectron. 2008, 23, 1819. 18406597 10.1016/j.bios.2008.02.021

[23] M. Lee , N. Zine , A. Baraket , M. Zabala , F. Campabadal , R. Caruso , M. G. Trivella , N. Jaffrezic‐Renault , A. Errachid , Sens. Actuators, B 2012, 175, 201. 10.1007/978-1-4939-0928-5_5 24908294

[24] M. Lee , A. Baraket , N. Zine , M. Zabala , F. Campabadal , R. Caruso , M. G. Trivella , N. Jaffrezic‐Renault , A. Errachid , in Cytokine Bioassays: Methods and Protocols (Ed: I. Vancurova ), Springer, New York, NY 2014. 10.1007/978-1-4939-0928-5_5 24908294

[25] N. Tuleuova , C. N. Jones , J. Yan , E. Ramanculov , Y. Yokobayashi , A. Revzin , Anal. Chem. 2010, 82, 1851. 20121141 10.1021/ac9025237

[26] G. D. McEwen , F. Chen , A. Zhou , Anal. Chim. Acta 2009, 643, 26. 19446060 10.1016/j.aca.2009.03.050 PMC2754797

[27] N. Tuleuova , A. Revzin , Cell Mol. Bioeng. 2010, 3, 337. 21170394 10.1007/s12195-010-0148-5 PMC2991185

[28] S. Farid , X. Meshik , M. Choi , S. Mukherjee , Y. Lan , D. Parikh , S. Poduri , U. Baterdene , C.‐E. Huang , Y. Y. Wang , P. Burke , M. Dutta , M. A. Stroscio , Biosens. Bioelectron. 2015, 71, 294. 25919809 10.1016/j.bios.2015.04.047

[29] J. Zhao , C. Chen , L. Zhang , J. Jiang , R. Yu , Biosens. Bioelectron. 2012, 36, 129. 22575639 10.1016/j.bios.2012.04.013

[30] R. Ohno , H. Ohnuki , H. Wang , T. Yokoyama , H. Endo , D. Tsuya , M. Izumi , Biosens. Bioelectron. 2013, 40, 422. 22917917 10.1016/j.bios.2012.07.052

[31] M. Varshney , Y. Li , Biosens. Bioelectron. 2009, 24, 2951. 19041235 10.1016/j.bios.2008.10.001

[32] P. V. Gerwen , W. Laureys , G. Huyberechts , M. D. Baeck , K. Baert , J. Suis , A. Varlan , W. Sansen , L. Hermans , R. Mertens , Proceedings of Int. Solid State Sensors and Actuators Conf. (Transducers '97) , IEEE, Piscataway, NJ, 1997.

[33] Z. Zou , J. Kai , M. J. Rust , J. Han , C. H. Ahn , Sens. Actuators, A 2007, 136, 518.

[34] Z. Yang , Z. Jian , X. Chen , J. Li , P. Qin , J. Zhao , X. a. Jiao , X. Hu , Biosens. Bioelectron. 2015, 63, 190. 25089816 10.1016/j.bios.2014.07.032

[35] S. Ding , S. R. Das , B. J. Brownlee , K. Parate , T. M. Davis , L. R. Stromberg , E. K. L. Chan , J. Katz , B. D. Iverson , J. C. Claussen , Biosens. Bioelectron. 2018, 117, 68. 29886188 10.1016/j.bios.2018.04.016

[36] K. M. Marr , B. Chen , E. J. Mootz , J. Geder , M. Pruessner , B. J. Melde , R. R. Vanfleet , I. L. Medintz , B. D. Iverson , J. C. Claussen , ACS Nano 2015, 9, 7791. 26106943 10.1021/acsnano.5b02124

[37] B. J. Brownlee , K. M. Marr , J. C. Claussen , B. D. Iverson , Sens. Actuators, B 2017, 246, 20.

[38] D. Liu , R. K. Perdue , L. Sun , R. M. Crooks , Langmuir 2004, 20, 5905. 16459608 10.1021/la049605p

[39] A. D. McNaught , A. Wilkinson , IUPAC Compendium of Chemical Terminology , Blackwell Scienctific Publications, Hoboken, NJ 1997.

[40] R. R. Moore , C. E. Banks , R. G. Compton , Anal. Chem. 2004, 76, 2677. 15144174 10.1021/ac040017q

[41] D. Salinas‐Torres , F. Huerta , F. Montilla , E. Morallón , Electrochim. Acta 2011, 56, 2464.

[42] W. Laureyn , P. Van Gerwen , J. Suls , P. Jacobs , G. Maes , Electroanalysis 2001, 13, 204.

[43] C. E. Banks , R. R. Moore , T. J. Davies , R. G. Compton , Chem. Commun. 2004, 21, 1804. 10.1039/b406174h 15306892

[44] C. E. Banks , T. J. Davies , G. G. Wildgoose , R. G. Compton , Chem. Commun. 2005, 56, 829. 10.1039/b413177k 15700054

[45] S. Hardeman , T. Nelson , D. Beirne , M. DeSilva , P. J. Hesketh , G. J. Maclay , S. M. Gendel , Sens. Actuators, B 1995, 24, 98.

[46] M. Zourob , S. Elwary , A. P. F. Turner , Principles of Bacterial Detection: Biosensors, Recognition Receptors and Microsystems, Springer, New York 2008.

[47] B. M. Szydlowska , C. C. Pola , Z. Cai , L. E. Chaney , J. Hui , R. Sheets , J. Carpenter , D. Dean , J. C. Claussen , C. L. Gomes , M. C. Hersam , ACS Appl. Mater. Interfaces 2024, 16, 25169. 38695741 10.1021/acsami.4c05264

[48] T. J. Kindt , R. A. Goldsby , B. A. Osborne , J. Kuby , Kuby Immunology, 6th ed. W. H. Freeman , New York 2007.

[49] J. Tate , G. Ward , Clin. Biochem. Rev. 2004, 25, 105. 18458713 PMC1904417

[50] Y. Zhang , B. Zhang , X. Ye , Y. Yan , L. Huang , Z. Jiang , S. Tan , X. Cai , Mater. Sci. Eng., C 2016, 59, 577. 10.1016/j.msec.2015.10.066 26652410

[51] M. S. Khalifeh , J. R. Stabel , Infect. Immun. 2004, 72, 1974. 15039317 10.1128/IAI.72.4.1974-1982.2004 PMC375184

[52] K. Parate , S. V. Rangnekar , D. Jing , D. L. Mendivelso‐Perez , S. Ding , E. B. Secor , E. A. Smith , J. M. Hostetter , M. C. Hersam , J. C. Claussen , ACS Appl. Mater. Interfaces 2020, 12, 8592. 32040290 10.1021/acsami.9b22183

[53] K. C. Januarie , M. Oranzie , U. Feleni , E. Iwuoha , Electrochim. Acta 2023, 463, 142825.

[54] H. A. Saputra , J. H. Chung , R. J. Kwon , K. A. Jannath , D.‐S. Park , Y.‐B. Shim , Sens. Actuators, B 2024, 398, 134739.

[55] I. A. M. Frias , N. Zine , M. Sigaud , P. Lozano‐Sanchez , M. Caffio , A. Errachid , Biosens. Bioelectron. 2023, 222, 114954. 36502717 10.1016/j.bios.2022.114954

[56] A. J. Bandodkar , I. Jeerapan , J.‐M. You , R. Nuñez‐Flores , J. Wang , Nano Lett. 2016, 16, 721. 26694819 10.1021/acs.nanolett.5b04549 PMC4713296

[57] A. A. Rowe , R. J. White , A. J. Bonham , K. W. Plaxco , JoVE 2011, 52, e2922. 10.3791/2922 PMC3197062 21673639

[58] L. Kong , W. Chen , Adv. Mater. 2014, 26, 1025. 24338697 10.1002/adma.201303432

[59] R. Hu , B. A. Cola , N. Haram , J. N. Barisci , S. Lee , S. Stoughton , G. Wallace , C. Too , M. Thomas , A. Gestos , M. E. d. Cruz , J. P. Ferraris , A. A. Zakhidov , R. H. Baughman , Nano Lett. 2010, 10, 838. 20170193 10.1021/nl903267n

